# Impact of Pediatric Dermatologic Conditions on Child and Parent Quality of Life

**DOI:** 10.7759/cureus.42068

**Published:** 2023-07-18

**Authors:** Mildred Min, Jasminder K Malhi, Cindy J Chambers, Raja Sivamani

**Affiliations:** 1 Dermatology, California Northstate University College of Medicine, Elk Grove, USA; 2 Anesthesiology, California Northstate University College of Medicine, Elk Grove, USA; 3 Dermatology, University of California Davis, Sacramento, USA; 4 Biological Sciences, California State University, Sacramento, USA

**Keywords:** quality of life, psoriasis, pediatric dermatology, eczema, acne vulgaris

## Abstract

Dermatologic conditions can confer a negative effect on pediatric patients and their caretakers. We aim to study the relationship between child and parent quality of life among various dermatoses to further understand the psychosocial impacts of dermatologic disease. We conducted a cross-sectional study of 100 pediatric patients (aged 7-18) and 98 parents who presented to the Pacific Skin Institute, Sacramento, CA, from November 2020 to January 2022. Patients and their parents were evaluated using the Children’s Dermatology Life Quality Index (CDLQI) and Family Dermatology Life Quality Index (FDLQI). The maximum score for both indices was 30, with a higher score indicating greater impairment on quality of life. From all the patients and parents identified for various dermatoses, FDLQI scores (mean, 7.8; n = 98) exceeded CDLQI scores (mean, 5.8; n = 100) in nearly every condition. Acne was the only diagnosis with greater CDLQI scores (mean, 9.2; n = 43) than FDLQI scores (mean, 8.8; n = 42). Psoriasis had the greatest difference between FDLQI scores (mean, 10.4; n = 9) and CDLQI scores (mean, 5.9; n = 9). Our study found that parents of children with dermatologic conditions often experience a greater impairment on quality of life compared to the patient. This is likely because parents are highly involved in the management of their child’s condition and are burdened with the costs associated with dermatoses. These findings call for a more holistic evaluation by clinicians and the expansion of resources for patients and their parents.

## Introduction

Dermatological conditions are frequently encountered in pediatric practice. Some of the most common pediatric skin conditions include atopic and contact dermatitis, birthmarks, and acne [1]. Especially in children, skin conditions are often accompanied by feelings of poor self-esteem and embarrassment, leading to a significant impact on quality of life [2-5]. Other symptoms caused by skin conditions could lead to the inability to wear certain clothes or difficulty focusing or sleeping [4]. There are several ways to assess for negative psychosocial effects, but the Dermatology Life Quality Index (DLQI) questionnaire has become one of the most widespread for the assessment of various dermatoses and their impact on several psychosocial domains of life [6,7]. The DLQI can assess specific domains pertaining to symptoms and feelings, personal relationship, sleep, treatment, school or holidays, and leisure [8]. 

One of the most studied pediatric skin conditions in relation to quality of life is acne. Acne, despite being a nonlife-threatening condition, has significant psychosocial impacts [9]. Studies have shown that acne in adolescents is highly associated with comorbidities such as anxiety and mood disorders [9]. Skin conditions affecting physical appearance, like acne, eczema, and psoriasis, also confer a negative impact on self-esteem due to increased incidences of bullying [10-13]. Eczema, one of the most common inflammatory dermatoses in children, is intensely pruritic, which can be debilitating in other domains of life such as sleep and leisure [14]. Pediatric patients with dermatological condition(s) usually experience numerous symptoms, which ultimately lead to a decreased quality of life and increased risk of psychological comorbidities [15]. 

Parents of children with skin conditions may also experience negative psychosocial consequences. For example, parents of children diagnosed with atopic dermatitis have reported the following lifestyle changes: increased household expenditure, reduced emotional and physical health, and less spare time and social life [16]. Parents play a large role in the management of their child’s skin condition, so understanding how they are affected can also impact the child’s wellness and adherence to treatment regimen(s) [17]. Children face many challenges as they develop their identity and self-image. Dermatological conditions can affect the way children feel, their relationships with others, the activities they choose to participate in, their school experience, and more [18]. Knowledge of the impact of skin disease on children and their caregivers is essential for appropriate and thorough management of their condition. This study aimed to evaluate the impact of dermatoses on the lives of children and to assess the burden of their diagnosis on their parent(s).

## Materials and methods

Ethics

This study was exempted by the UC Davis Institutional Review Board, and informed consent was waived for this anonymous survey. Licensure for Children’s DLQI (CDLQI) and Family DLQI (FDLQI) implementation was obtained.

Study design

We conducted a cross-sectional study utilizing CDLQI and FDLQI questionnaires. Responses were collected anonymously from November 2020 to January 2022 at the Pacific Skin Institute, Sacramento, CA. We surveyed 100 patients aged seven to 18, diagnosed with a dermatologic condition or suffering from an undiagnosed skin condition, and 98 parents over 18 years old. The studied conditions and their respective pediatric sample sizes are acne (46), eczema (26), psoriasis (nine), undiagnosed (six), wart (four), nevi (three), port-wine stain (two), vitiligo (two), cyst (one), pityriasis rosea (one), keratosis pilaris (one), cafe-au-lait spots (one), and molluscum contagiosum (one). The sum of the individual pediatric sample sizes exceeds 100 since some patients were diagnosed with multiple conditions. Exclusion criteria included children younger than seven years or older than 18 years, parents younger than 18 years, and children and parents unable to read, write, and speak English. The diagnoses and patient age were also collected via survey. CDLQI and FDLQI scores were calculated using Excel and stratified by diagnosis. 

Study measures

The maximum score for CDLQI and FDLQI responses is 30 points, with greater scores indicating greater impairment on quality of life. For the CDLQI, total scores of zero to one, two to six, seven to 12, 13-18, and 19-30 correspond, respectively, to no effect, small effect, moderate effect, very large effect, and extremely large effect on child life quality; FDLQI scores are not categorized in a similar manner. CDLQI scores were then stratified and calculated by domains (“symptoms and feelings,” “personal relationships,” “leisure,” “school or holidays,” “sleep,” and “treatment”) to determine the impact on quality of life within specific aspects of the patient’s life.

## Results

The study population included 100 pediatric patients with a dermatological condition and 98 parents. The pediatric participants were aged seven to 18, and all participants were from the Pacific Skin Institute, Sacramento, CA. The most common skin conditions studied in descending order included acne, eczema, and psoriasis, which composed 46%, 26%, and 9% of the participants, respectively. The average CDLQI and FDLQI scores stratified by diagnosis are outlined in Table 1.

**Table 1:** Average CDLQI and FDLQI scores by diagnosis CDLQI, Children’s Dermatology Life Quality Index; FDLQI, Family Dermatology Life Quality Index

Diagnosis	Average CDLQI score	Average FDLQI score
Eczema	9.38	10.36
Acne	9.20	8.8
Psoriasis	5.89	10.44
Undiagnosed	6.83	6.8
Cyst	9	13
Wart	4	6.75
Port-wine stain	1.5	3
Vitiligo	4.5	4.5
Nevi	3.33	4.33
Pityriasis rosea	5	10
Keratosis pilaris	8	12
Cafe-au-lait spots	2	4
Molluscum contagiosum	7	7

CDLQI scores ranged from 1.5 to 9.38 (mean, 5.8; n = 100), with a higher score indicating greater impairment on quality of life. CDLQI scores were the greatest among patients diagnosed with the following in descending order: eczema, acne, cyst, keratosis pilaris, molluscum contagiosum, and undiagnosed. These diagnoses fall into the category of moderate effect on child quality of life based on the CDLQI scores. CDLQI scores, in descending order, for psoriasis, pityriasis rosea, vitiligo, wart, nevi, cafe-au-lait spots, and port-wine stain fall into the category of small effect on child quality of life. FDLQI scores ranged from 3 to 12 (mean, 7.8; n = 98). FDLQI scores revealed that parent quality of life was most impacted for those with children diagnosed with cysts, followed in descending order by keratosis pilaris, psoriasis, eczema, pityriasis rosea, acne, molluscum contagiosum, undiagnosed, wart, vitiligo, nevi, cafe-au-lait spots, and port-wine stain (Table 1). CDLQI scores stratified by domains are presented in Table 2.

**Table 2:** CDLQI scores stratified by domains CDLQI, Children’s Dermatology Life Quality Index

Diagnosis	Symptoms and feelings	Personal relationships	Leisure	School or holidays	Sleep	Treatment
Eczema	3.0	1.0	2.7	0.8	0.8	1.2
Acne	3.0	1.5	1.9	0.8	0.6	1.3
Psoriasis	1.9	0.8	1.4	0.4	0.6	0.8
Undiagnosed	2.5	1.0	1.2	0.3	0.7	1.2
Cyst	2.0	1.0	5.0	0	0	1.0
Wart	1.3	0.8	1.0	0.3	0.3	0.5
Port-wine stain	1.0	0.5	0	0	0	0
Vitiligo	3.0	0	1.5	0	0	0
Nevi	1.0	0.3	1.0	0.3	0.3	0.3
Pityriasis rosea	2.0	0	2.0	0	0	1.0
Keratosis pilaris	1.0	2.0	3.0	1.0	0	1.0
Cafe-au-lait spots	1.0	0	1.0	0	0	0
Molluscum contagiosum	1.0	2.0	3.0	1.0	0	0
Average scores	1.79	0.79	1.77	0.38	0.30	0.64

Average CDLQI domain scores ranged from 0.30 to 1.79. The average scores for “symptoms and feelings” (max score = 6) were the highest, followed in descending order by “leisure” (max score = 9), “personal relationships” (max score = 6), “treatment” (max score = 3), “school or holidays” (max score = 3), and “sleep” (max score = 3). Higher scores indicated a higher degree of impact on the domain being tested. 

## Discussion

We have studied the psychosocial impacts of pediatric skin conditions by demonstrating their effects on child and parent quality of life, stratified by diagnosis. Previous studies have analyzed child and/or parent quality of life for specific pediatric skin diagnoses [1-5]. Our study is unique in that we have quantified the effect of a wide number of pediatric skin conditions on overall life quality and specific CDLQI domains. 

Interestingly, some trends and deviations were seen in the results obtained. The mean FDLQI scores for most of the studied skin conditions were similar to or higher than their respective CDLQI scores, indicating a greater impact on parent quality of life rather than child quality of life. These findings are related to the psychosocial impacts that come with caring for a child with a dermatologic condition. For instance, factors such as financial expenditure, time management, and associated impacts on the mental and physical health of the parents may be significant. However, acne patients were an exception to this trend in that the average CDLQI score was greater than the average FDLQI score. Another study also displayed the same trend of acne patients experiencing a greater impact on their quality of life in comparison to family [19]. However, comorbid conditions in acne patients, such as anxiety and depression, could also lead to a negative impact on their quality of life [19]. For these reasons and more, international organizations such as The European Academy of Dermatology and Venereology Task Force on Quality of Life recommend the use of health-related quality of life assessments as part of acne management [20]. The significant cosmetic burden that acne patients experience is a major factor in their greater impact on quality of life in comparison to parents in this study. Moreover, patients with psoriasis had the greatest difference between FDLQI and CLDQI scores. More research is necessary to investigate the large gap between parents and children with psoriasis. Finally, the average FDLQI scores were exceedingly greater than the average CDLQI scores for cyst, pityriasis rosea, and keratosis pilaris as well though those results may be skewed by the small sample size for those diagnoses. 

Pediatric skin conditions confer a negative impact on both the child and parent quality of life. As such, it is important for physicians to consider psychosocial impacts and approach the patient holistically. For instance, since child “symptoms and feelings” appear to be most significantly impacted in our sample of patients, physicians can address the patient’s negative feelings in addition to physical symptoms. By addressing the presence of and the root of these negative feelings, physicians can help to alleviate such emotions accordingly through education, encouragement, and recommendations for ancillary services such as support groups [21]. The other CDLQI domains and parent quality of life can be addressed similarly. Addressing parent quality of life is important as well, considering the significant role parents play in the management of the child’s skin condition at home. Dermatological skin conditions in children, irrespective of the type, impair the quality of life of the child and their caregiver. 

Limitations

There are some limitations to this study. A large number of the participants were diagnosed with eczema or acne, with fewer children diagnosed with the other studied conditions. Therefore, the statistics may be skewed for the less prevalent conditions and may require an expanded follow-up. Also, patients with common conditions such as hidradenitis suppurativa were not present in this study, so those diseases are not necessarily subject to the results. Additionally, participants were recruited from one outpatient clinic in Sacramento, CA. Thus, the results of the study cannot be generalized for patients elsewhere. In future studies, it would be interesting to perform similar research on a larger scale in regards to location and sample size.

## Conclusions

Pediatric skin conditions are common and have physical and psychosocial implications for the patient and their parents. In addition to the physical symptoms of their skin condition, children experience feelings of shame or embarrassment, disruptions in leisure activities, difficulty in personal relationships, impact on schoolwork or holiday enjoyment, and impairment of sleep. Parents are also impacted by their child’s skin condition. In fact, this study shows that parents of children with dermatologic conditions often experience a greater impairment on quality of life when compared to the patient. Dermatological conditions are not just a disease of the skin. Understanding the psychosocial impact of a skin condition should be a part of the holistic dermatologic evaluation. Additional support by the physician, such as recommendations for support groups and access to mental health professionals, should also be considered.

## References

[1] Castelo-Soccio L, McMahon P (2017). Pediatric dermatology. J Clin Aesthet Dermatol.

[2] Vivar KL, Kruse L (2018). The impact of pediatric skin disease on self-esteem. Int J Womens Dermatol.

[3] Gånemo A, Svensson A, Lindberg M, Wahlgren CF (2007). Quality of life in Swedish children with eczema. Acta Derm Venereol.

[4] Olsen JR, Gallacher J, Finlay AY, Piguet V, Francis NA (2016). Quality of life impact of childhood skin conditions measured using the Children's Dermatology Life Quality Index (CDLQI): a meta-analysis. Br J Dermatol.

[5] Weber MB, Lorenzini D, Reinehr CP, Lovato B (2012). Assessment of the quality of life of pediatric patients at a center of excellence in dermatology in southern Brazil. An Bras Dermatol.

[6] Chernyshov PV (2019). The evolution of quality of life assessment and use in dermatology. Dermatology.

[7] Chen SC (2012). Health-related quality of life in dermatology: introduction and overview. Dermatol Clin.

[8] Finlay AY, Khan GK (1994). Dermatology Life Quality Index (DLQI)--a simple practical measure for routine clinical use. Clin Exp Dermatol.

[9] Stamu-O'Brien C, Jafferany M, Carniciu S, Abdelmaksoud A (2021). Psychodermatology of acne: psychological aspects and effects of acne vulgaris. J Cosmet Dermatol.

[10] Gieler U, Gieler T, Kupfer JP (2015). Acne and quality of life - impact and management. J Eur Acad Dermatol Venereol.

[11] Kaymak Y, Taner E, Taner Y (2009). Comparison of depression, anxiety and life quality in acne vulgaris patients who were treated with either isotretinoin or topical agents. Int J Dermatol.

[12] Awad SM, Morsy H, Sayed AA, Mohamed NA, Ezzat GM, Noaman MM (2018). Oxidative stress and psychiatric morbidity in patients with facial acne. J Cosmet Dermatol.

[13] Capucci S, Hahn-Pedersen J, Vilsbøll A, Kragh N (2020). Impact of atopic dermatitis and chronic hand eczema on quality of life compared with other chronic diseases. Dermatitis.

[14] Fishbein AB, Silverberg JI, Wilson EJ, Ong PY (2020). Update on atopic dermatitis: diagnosis, severity assessment, and treatment selection. J Allergy Clin Immunol Pract.

[15] Goyal S, Sajid N, K Nayak SU, Husain S (2021). CDLQI-based assessment of skin disorders among children: a study from Northern India. Indian J Dermatol.

[16] Marciniak J, Reich A, Szepietowski JC (2017). Quality of life of parents of children with atopic dermatitis. Acta Derm Venereol.

[17] Ezzedine K, Shourick J, Merhand S, Sampogna F, Taïeb C (2020). Impact of atopic dermatitis in adolescents and their parents: a French Study. Acta Derm Venereol.

[18] Lewis-Jones MS, Finlay AY (1995). The Children's Dermatology Life Quality Index (CDLQI): initial validation and practical use. Br J Dermatol.

[19] Martínez-García E, Arias-Santiago S, Herrera-Acosta E, Affleck A, Herrera-Ceballos E, Buendía-Eisman A (2020). Quality of life of cohabitants of people living with acne. Acta Derm Venereol.

[20] Marron SE, Chernyshov PV, Tomas-Aragones L (2019). Quality-of-life research in acne vulgaris: current status and future directions. Am J Clin Dermatol.

[21] Salek MS, Jung S, Brincat-Ruffini LA, MacFarlane L, Lewis-Jones MS, Basra MK, Finlay AY (2013). Clinical experience and psychometric properties of the Children's Dermatology Life Quality Index (CDLQI), 1995-2012. Br J Dermatol.

